# Linking bacterial and fungal assemblages to soil nutrient cycling within different aggregate sizes in agroecosystem

**DOI:** 10.3389/fmicb.2022.1038536

**Published:** 2022-11-14

**Authors:** Shan Zhang, Wanjin Hu, Yue Xu, Hui Zhong, Zhaoyu Kong, Lan Wu

**Affiliations:** School of Life Sciences, Key Laboratory of Poyang Lake Environment and Resource Utilization, Ministry of Education, Nanchang University, Nanchang, China

**Keywords:** agricultural soil, deterministic processes, microbial activities, soil aggregate, stochastic processes

## Abstract

Soil aggregates provide spatially heterogeneous microhabitats that support the coexistence of soil microbes. However, there remains a lack of detailed assessment of the mechanism underlying aggregate-microbiome formation and impact on soil function. Here, the microbial assemblages within four different aggregate sizes and their correlation with microbial activities related to nutrient cycling were studied in rice fields in Southern China. The results show that deterministic and stochastic processes govern bacterial and fungal assemblages in agricultural soil, respectively. The contribution of determinism to bacterial assemblage improved as aggregate size decreased. In contrast, the importance of stochasticity to fungal assemblage was higher in macroaggregates (>0.25 mm in diameter) than in microaggregates (<0.25 mm). The association between microbial assemblages and nutrient cycling was aggregate-specific. Compared with microaggregates, the impacts of bacterial and fungal assemblages on carbon, nitrogen, and phosphorus cycling within macroaggregates were more easily regulated by soil properties (i.e., soil organic carbon and total phosphorus). Additionally, soil nutrient cycling was positively correlated with deterministic bacterial assemblage but negatively correlated with stochastic fungal assemblage in microaggregates, implying that bacterial community may accelerate soil functions when deterministic selection increases. Overall, our study illustrates the ecological mechanisms underlying the association between microbial assemblages and soil functions in aggregates and highlights that the assembly of aggregate microbes should be explicitly considered for revealing the ecological interactions between agricultural soil and microbial communities.

## Introduction

Bacteria and fungi are essential microorganisms that perform critical ecological roles in soil structure formation, and nutrient cycling (e.g., organic matter decomposition, nitrification, denitrification, and phosphorous solubilization) (Tedersoo et al., 2014; Dai et al., 2021; Jiao et al., 2021). The role of microorganisms in soil ecosystems is associated with shifts in community diversity and composition (Liu et al., 2021; Wan et al., 2021; Han et al., 2022). However, the fundamental mechanisms that underpin microbial assemblages have not been fully explored, although understanding these mechanisms is vital for preserving soil function and ecosystem services.

Both deterministic and stochastic processes may affect microbial assemblages and play essential roles in maintaining community diversity and composition (Stegen et al., 2013; Zhou and Ning, 2017). Deterministic processes influence fitness of microbial communities and then alter species composition and abundance (Powell et al., 2015). The increase in determinism leads to a convergent community (Xu et al., 2020). Conversely, stochastic processes can increase community variation or turnover (Lan et al., 2020; Osburn et al., 2021) and result in unpredictable changes in community structure (Zhou and Ning, 2017). In general, deterministic and stochastic processes can jointly affect microbial communities in most cases (Zhang et al., 2016; Luan et al., 2020), but the relative contributions of specific ecological processes may be “scale-dependence” (Aiello-Lammens et al., 2017; Catano et al., 2021). For example, the assemblage of bacteria changed from homogeneous selection at local scales to variable selection at regional scales (Wisnoski and Lennon, 2021). Xue et al. (2021) found that bacterial assemblage was deterministic at a centimeter scale but changed from variable selection to homogenous selection as the sample size increased. Nevertheless, given that soil microbes are intensively distributed, and microbial interactions could occur at the micron scale (Raynaud and Nunan, 2014), the microbial assemblages may be detected on more minor scales. Therefore, considering assembly processes in more dimensions is necessary, particularly in microbial communities at microscopic scales.

Additionally, the variations in the assembly processes substantially affect the soil function by determining the shifts in microbial structure (Stegen et al., 2016). Deterministic processes of variable selection may putatively favor a well-adapted community concerning prevailing conditions, resulting in increased microbial metabolic capacity and stable ecosystem function (Graham et al., 2016; Graham and Stegen, 2017). On the contrary, stochastic processes may mediate adverse impacts from high rates of environmental changes on ecosystems and maintain diverse soil functions (Zhou et al., 2013; Liu et al., 2021). Carbon mineralization is most elevated when selective pressures are maximized, but dispersal limitation is minimized (Luan et al., 2020). Moreover, the extent to which microbial assemblages impact soil functions is contingent on myriad spatiotemporal dynamics, including the geographic distance, the rate of environmental change, and historical abiotic conditions (Knelman and Nemergut, 2014; Graham et al., 2016; Liu et al., 2021). Consequently, exploring the association between microbial assemblages and ecosystem functions is sparse and challenging.

Aggregates are the fundamental structural units of soil and can create spatially heterogeneous microhabitats for microbiome and ecological processes (Six et al., 2004; Wang et al., 2018). The size and stability of aggregates can impact the microbial communities by altering environmental conditions (e.g., organic substrates, total porosity, and air-and water-filled pore spaces) and biotic interactions from the surrounding bulk soil or neighboring aggregates (Li et al., 2019; Han et al., 2021). Differences in nutrient availability and physical properties in various aggregate sizes impose selective pressures on microorganisms and are the primary factor governing microbial abundances and diversities at the fine scale (Smith et al., 2014; Gupta and Germida, 2015). However, studies so far have mainly focused on variations in community structure (Wilpiszeski et al., 2019; Hou et al., 2021; King et al., 2021), whereas aggregate-microbiome assembly, particularly the relationship with soil function, is still poorly understood.

Rice is widely cultivated in China. However, excessive fertilizer application and irrational agricultural management have intensified soil compaction, which in turn has impacted the stability of soil aggregates and hence plant nutrient transfer (Ye et al., 2015; Han et al., 2021; Zheng et al., 2022). This situation motivated us to investigate microbial distribution patterns and decipher their potential function in an agroecosystem at aggregate scales. Thus, we carried out an investigation on the soil bacterial and fungal communities in rice fields in Southern China. We aimed to (1) investigate the relative importance of deterministic and stochastic processes in shaping microbial communities within different aggregate sizes in agricultural soil; and (2) decipher the effects of bacterial and fungal assemblages on soil carbon, nitrogen, and phosphorus cycling.

## Materials and methods

### Study sites and soil sampling

Soil samples were collected in early May 2017 from four regions cultivated with rice on three islands in Nanchang (Jiangxi Province, China, Supplementary Figure S1A). These regions were more than 5 km apart and the moisture condition of the sites could be described as wet but not flooded during sampling. Two central cropping management systems were in use: the single-season cropping system (RS), which cultivated rice once a year, and the double-season cropping system (RD), which cultivated rice twice a year. Chemical fertilizer in the form of NPK (about 395–425 kg ha^−1^, N: 195–200 kg ha^−1^, P: 90–100 kg ha^−1^, K: 110–125 kg ha^−1^) was applied during the rice growing cycle. The mean annual temperature was 17.5°C, with an average yearly rainfall of 1,470 mm and 69.4% occurring from April to September. According to the International Standard for Soil Texture Classification (Christensen, 1986), the soils in the study area are paddy soil and classified as clay loam (average sand: 50.39%, silt: 33.69%, clay: 15.92%, determined by the hydrometer method (Beretta et al., 2014)).

Sample sites for RS and RD were chosen from adjacent areas of each rice-growing region (Supplementary Figure S1B). Three independent replicating plots (10 × 10 m^2^) were established at each site, and neighboring plots partitioned through ridges left by agricultural practice. Plots were divided into four subplots, each with an area of 5 × 5 m^2^, and five soil cores (2.5 cm in diameter, 20 cm in depth) were obtained from each using a soil auger. After removing large rocks and roots, the 20 soil cores from each plot were pooled to obtain a representative bulk soil sample. At the same time, aggregate samples were collected from undisturbed soil in each subplot (Supplementary Figure S1C). Five blocks (20 × 20 × 20 cm^3^) were collected in each subplot, and these 20 soil blocks were placed on ice and transported to the laboratory with bulk soils.

### Soil aggregate fractionation

Aggregate fractionation was performed using the “optimal moist-sieving procedure” (Bach and Hofmockel, 2014; Wang et al., 2021). Briefly, when soil blocks were dried to “optimum moisture” (approximately 10–14% gravimetric water content) at 4°C, they were gently broken up along their natural fracture planes. All broken soils taken from one plot were evenly mixed as one sample and passed through an 8 mm sieve to remove large roots and rocks. The samples were then placed on sterile sieves of 5 mm, 2 mm, and 0.25 mm and sieved at a rate of 30 times per minute for 2 min by a mechanical shaker (Retsch, AS 200 basic, Haan, Germany). Four fractions were collected for each sample: >5 mm, 2–5 mm, 0.25–2 mm and < 0.25 mm (Supplementary Figure S1D). A total of 120 samples (2 rice cropping systems × 4 sites × 3 replicates × (4 aggregate fractions +1 bulk soil)) were thus obtained. Samples of bulk soil and each aggregate fraction were divided into three portions: one portion was stored at-80°C for DNA extraction, one was kept at 4°C to measure microbial biomass carbon (MBC) and nitrogen (MBN), carbon mineralization rate (Cmin), potential nitrification rate (PNR) and extracellular enzymes within 1 week, and the third was air-dried prior to analysis of physicochemical properties.

### Soil physicochemical properties and microbial activities

Mean weight diameter (MWD) and geometric mean diameter (GMD) were calculated to represent the stability of soil aggregates as follows:


$$MWD=\overset{n}{\underset{i=1}{\sum }}\mathrm{Xi}\times Wi$$

$$GMD=EXP\overset{n}{\underset{i=1}{\sum }}Wi\times \ln\left(\mathrm{Xi}\right)$$

Where *i* represents the collected fraction, *Wi* is the ratio of the weight fraction of the *i*-th size aggregates to the total soil sample, and *Xi* is the average diameter (mm) of each fraction (Guo et al., 2022).

Soil pH was measured in a soil-water suspension (1:2.5 w/v). Soil organic carbon (SOC), total nitrogen (TN) and total phosphorus (TP) were evaluated by the K_2_CrO_7_/H_2_SO_4_ oxidation procedure, the micro-Kjeldahl method and the phosphomolybdic acid blue color method, respectively (Liu, 1996).

Eight microbial activities involved in nutrient cycling were quantified, including C-cycling (i.e., microbial biomass carbon (MBC), carbon mineralization rate (Cmin), β-D-glucosidase (Bglu), β-D-xylanase (Bxyl), polyphenol oxidase (Poxi) and peroxidase (Pero)), N-cycling (microbial biomass nitrogen (MBN), potential nitrification rate (PNR) and N-acetyl glucosaminidase (NAG)) and P-cycling (phosphatase (Phos)). The chloroform fumigation-extraction method was applied to measure MBC and MBN (Vance et al., 1987; Domeignoz-Horta et al., 2020). A total organic carbon/nitrogen analyzer (TOC-VC/TN, Shimadzu, Kyoto, Japan) was used to measure the contents. An infrared gas analyser (Li 820, Licor, United States) connected to the soil culture system was used to measure Cmin (Wang et al., 2016). PNR was measured as described by Qian et al. (2017). The activity of soil enzymes was assayed using the protocol according to Hu et al. (2021). In brief, four hydrolases (Bglu, Bxyl, NAG, Phos) were assayed using 4-methylumbelliferyl (MUB) as substrate, and two oxidases (Poxi and Pero) were determined using 3, 4-dihydroxy-L-phenylalanine (L-DOPA) as substrate. Enzyme activity was measured using a Varioskan Flash spectrophotometer (Thermo Fisher Scientific, USA) and expressed as nmol h^−1^ g^−1^ dry mass soil units.

### DNA extraction and high-throughput sequencing

DNA was extracted from 0.25 g samples using the MoBio Powersoil DNA Isolation kit (MOBIO Laboratories, Inc., USA) according to manufacturer instructions. The concentration and purity of extracted DNA were quantified using SimpliNano (Biochrom, Berlin, Germany). The 16S rRNA gene of bacteria with the primers 338F and 806R (Finkel et al., 2020) and the internal transcribed spacer (ITS) region of fungi with the primers ITS5F and ITS2R (Jin et al., 2019) were sequenced by the Illumina Miseq platform (PE300) paired-end analysis (Personal Biotechnology Co., Ltd. Shanghai, China). All pyrosequencing reads were deposited in the National Center for Biotechnology Information (NCBI) Sequence Read Archive with accession PRJNA860287 and PRJNA860312, respectively.

The sequencing data were processed using Quantitative Insights into Microbial Ecology (QIIME2) (Uzan-Yulzari et al., 2021). In brief, raw data were cut using Trimmomtic-0.36 to remove ambiguous and low-quality readings (Bolger et al., 2014). Sequence splicing was performed using FLASH 1.2.11 (Magoč and Salzberg, 2011). Then primers and chimeric sequences were identified and removed using cutadapt 1.8 and Usearch 9.2, respectively (Edgar, 2010). Operational taxonomic units (OTUs) were clustered using Usearch 9.2 with a threshold of no less than 97% (Thiergart et al., 2020). Taxonomy was assigned against the SILVA 130 and UNITEITS reference database for bacterial 16S rRNA OTUs and fungal ITS OTUs, respectively (Quast et al., 2012).

### Statistical analysis

Statistical analyses were performed using IBM SPSS Statistics (version 26.0) and R software (version 4.1.1) unless otherwise mentioned. The differences in soil physicochemical properties, microbial activities, and alpha diversities within different aggregate sizes were evaluated by one-way ANOVA. The Shapiro–Wilk test and Levene’s test checked normality and homogeneity. Post-hoc comparisons of normal and non-normal distribution variables were assessed using Tukey HSD and Kruskal-Wallis test, respectively. Principle coordinates analysis (PCoA) based on Bray–Curtis dissimilarity was performed to evaluate the differences of microbial communities using the function “*cmdscale*” in the “*vegan*” package. Permutational multivariate ANOVA (PERMANOVA) was used to assess variations in microbial communities between groups.

Null model analysis was performed to quantify the ecological processes of bacterial and fungal assemblages within aggregates according to Stegen et al. (2013). Beta-nearest taxon index (βNTI) and Bray-Curtis-based Raup-Crick (RCbray) were calculated to quantify the contribution of deterministic and stochastic processes. |βNTI| > 2 indicated deterministic dominant processes (variable (βNTI >2) or homogeneous selection (βNTI < −2)). |βNTI| < 2 signified stochastic dominated processes; in this case, RCbray >0.95 indicated dispersal limitation, RCbray < −0.95 indicated homogenizing dispersal, and |RCbray| < 0.95 indicates undominated processes, mostly attributable to weak dispersal and selection, diversification, and drift (Zhou and Ning, 2017).

Niche breadth (B) was calculated to further explore the relative importance of deterministic and stochastic processes using the “niche breadth” function in package “*spaa*.” The community-level niche breadth was calculated as the average B values for all taxa in one community. The microbial communities with a broader niche represent more metabolically flexible at the community level (Zhang et al., 2018).

Co-occurrence patterns were analyzed to explore the potential roles of bacterial and fungal interactions within the microbial assemblages using network analysis (Chen et al., 2018). OTUs with relative abundance <0.01% were removed to reduce the complexity of networks. A similarity threshold of 0.80 was used to construct networks in this study, and only edges with adjusted *p* < 0.01 were retained (Jeewani et al., 2020). Mean path length, graph density, network diameter, the mean clustering coefficient, mean connectivity, and modularity were calculated with the “*igraph*” package to describe the complex pattern of interrelationships of bacterial and fungal communities. Network images were then visualized using Gephi software (version 0.9.2).

Mantel test was used to assess the interaction between soil physicochemical properties, characteristics of microbial interaction network (proportion of negative correlation, PNC) and microbial assemblages (βNTI) in the “*ecodist*” package (Mo et al., 2021). Random forest (RF) analysis was used to quantitatively illustrate the critical predictors of soil microbial activities, including physicochemical variables (pH, SOC, TN, TP, C:N and N:P), bacterial and fungal communities (bacterial and fungal assemblages (βNTI), diversities (Shannon index) and compositions (Bray–Curtis dissimilarity)), using the package “*randomForest*.”

Partial least squares structural equation modeling (PLS-SEM) was constructed to evaluate the direct and indirect relationships among physicochemical variables, bacterial and fungal communities, as well as soil nutrient cycling using the package “*plspm*” (Jiang et al., 2021). The first step in PLS-SEM required establishing an *a priori* model of the known variables, bacterial and fungal assemblages, and microbial activities based on the results of the Mantel test and RF analysis. Then we excluded the predictors of poor fitting to the model and established a unified structural equation modeling the data from each aggregate fraction. Standardized path coefficients were presented only when the significant level was less than 0.05.

## Results

### Soil physicochemical properties and microbial activities

**Figure 1:**
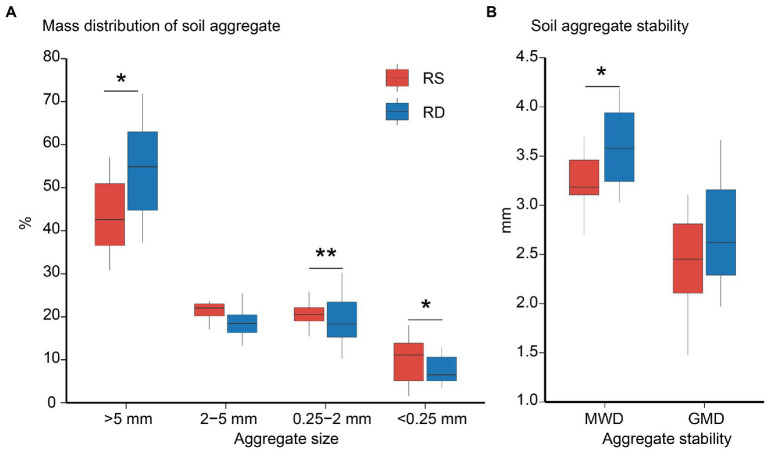
The mass distribution **(A)** and stability **(B)** of soil aggregate under single-season (RS) and double-season (RD) cropping systems. Significant differences at *p* < 0.05 is showed only. The asterisks indicate a significant difference between single-season and double-season cropping systems (*p < 0.05, **p < 0.01). MWD: Mean weight diameter; GMD: geometric mean diameter.

Compared with RS, the stability of soil aggregates was improved under RD by increasing the proportion of >5 mm aggregate and decreasing the ratios of 0.25–2 mm and < 0.25 mm aggregates (Figure 1, *p* < 0.05).

The contents of SOC and TN did not vary across aggregate sizes under RS or RD. Still, they were significantly higher under RD than RS within both bulk soil and aggregates (Table 1; Supplementary Figures S2B,C). TP was affected considerably by aggregate size (*R*^2^ = 0.155, *p* < 0.001) rather than cropping system (Table 1). The concentration of TP increased slightly from >5 mm (0.58 g kg^−1^) to 2–5 mm (0.71 g kg^−1^) aggregates and after that declined under RS while declining as aggregate sizes decreased under RD (Supplementary Figure S2D). Additionally, the N:P ratio gradually increased with decreasing aggregate size under RD (*p* < 0.05), but no marked changes in aggregate size were observed under RS (Table 1; Supplementary Figure S2F).

**Table 1:** Permutational multivariate ANOVA showing the effects of soil aggregate size and cropping system on soil physicochemical properties, microbial activities, and communities.

	Aggregate size		Cropping system		Interaction	
	*F*	*R* ^2^	*F*	*R* ^2^	*F*	*R* ^2^
**Soil properties**	1.242	0.042	1.498	0.013	0.360	0.012
pH	44.915	0.606***	3.272	0.011	0.829	0.011
SOC	0.513	0.018	1.713	0.015*	0.198	0.007
TN	0.538	0.019	2.446	0.021*	0.177	0.006
TP	5.144	0.155***	0.559	0.004	0.439	0.013
C:N	2.831	0.086*	0.594	0.005	2.523	0.076*
N:P	2.116	0.069*	2.355	0.019	0.352	0.012
**Microbial activities**	89.559	0.732***	12.415	0.025***	2.163	0.018
MBC	17.217	0.361***	1.242	0.007	2.694	0.056*
MBN	28.238	0.461***	15.077	0.061***	1.792	0.029
Cmin	4.875	0.139***	1.115	0.008	2.492	0.071
PNR	14.527	0.339***	1.498	0.009	0.499	0.012
Bglu	434.410	0.887***	82.660	0.042	6.940	0.014***
Bxyl	32.931	0.530***	3.736	0.015*	0.827	0.013
NAG	55.520	0.667***	0.210	0.001	0.205	0.002
Phos	134.466	0.750***	46.423	0.065***	5.748	0.032***
Poxi	5.219	0.154**	3.025	0.022	0.464	0.014
Pero	40.053	0.564***	1.269	0.004	3.117	0.044*
Bacterial Chao 1	0.569	0.019	2.958	0.025*	1.264	0.042
Bacterial Shannon	2.730	0.078	10.519	0.081***	0.987	0.029
Fungal Chao 1	0.613	0.021	3.787	0.032*	0.617	0.021
Fungal Shannon	1.014	0.034	0.304	0.003	1.644	0.054
Bacterial composition	1.279	0.041*	5.302	0.043***	0.762	0.025
Fungal composition	1.816	0.058***	4.199	0.033***	1.100	0.035
Bacterial βNTI	3.291	0.097**	5.839	0.043**	1.549	0.046
Fungal βNTI	0.586	0.019	1.770	0.014	2.684	0.086*
PNC	1.683	0.032*	3.253	0.073**	1.051	0.026

The asterisks indicate a significant influence (*p < 0.05, **p < 0.01, ***p < 0.001).

SOC, Soil organic carbon; TN, Total nitrogen; TP, Total phosphorus; C:N, The ratio of soil organic carbon and nitrogen; N:P, The ratio of soil nitrogen and phosphorus; MBC, Microbial biomass carbon; MBN, Microbial biomass nitrogen; Cmin, Carbon mineralization rate; PNR, Potential nitrification rate; Bglu, β-D-glucosidase; Bxyl, β-D-xylanase; NAG, N-acetyl glucosaminidase; Phos, Phosphatase; Poxi, Polyphenol oxidase; Pero, Peroxidase. βNTI, Beta-nearest taxon index; PNC, the proportion of negative correlation.

PERMANOVA, where F is the relative distance between cluster centroids, revealed that soil aggregate size (*R*^2^ = 0.732, *p* < 0.001) yielded a substantial impact on the microbial activities, while cropping system (*R*^2^ = 0.025, *p* < 0.001) had less of an influence (Table 1). The maximum contents of MBC and MBN were observed within 0.25–2 mm aggregate (MBC: 441.70 mg kg^−1^, MBN: 21.19 mg kg^−1^) under both RS and RD among aggregates (*p* < 0.05), but the minimum values were found within >5 mm fraction (MBC: 328.08 mg kg^−1^, MBN: 19.55 mg kg^−1^, *p* < 0.05, Supplementary Figures S3A,B). Cmin increased significantly with decreasing aggregate sizes under RS (*p* < 0.05), but no marked change was observed under RD (Supplementary Figure S3C). PNR was significantly higher within the >5 mm (1.11 mg NO_3_^−^-N kg^−1^ h^−1^) and < 0.25 mm aggregates (1.16 mg NO_3_^−^-N kg^−1^ h^−1^) than within the 2–5 mm (0.58 mg NO_3_^−^-N kg^−1^ h^−1^) and 0.25–2 mm aggregates (0.56 mg NO_3_^−^-N kg^−1^ h^−1^) under RS (*p* < 0.05), while it declined sharply across aggregate sizes under RD (*p* < 0.05, Supplementary Figure S3D). Phos and Poxi were highest in 2–5 mm aggregate either under RS or RD but lowest in >5 mm and < 0.25 mm, respectively (*p* < 0.05, Supplementary Figures S3H,J). In addition, although the two cropping systems had no effects on microbial activities, including MBC, MBN, Cmin, Bglu, Bxyl, NAG, Phos, Phox and Pero in bulk soil, all measured microbial activities except for Phos were greater under RD than RS across each fraction of aggregates (*p* < 0.05, Supplementary Figure S3).

### Diversities and compositions of bacterial and fungal communities

**Figure 2:**
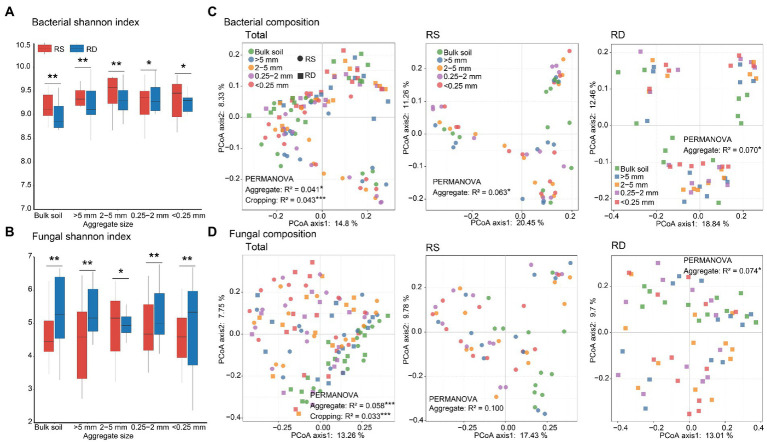
Differences in microbial diversities and compositions across four aggregate sizes under single-season (RS) and double-season (RD) cropping systems. Bacterial **(A)** and fungal **(B)** diversities across different aggregate sizes. Principle coordinates analysis for bacterial **(C)** and fungal **(D)** compositions across different aggregate sizes. The asterisks indicate a significant difference between single-season and double-season cropping systems (*p < 0.05, **p < 0.01).

The alpha diversities of bacteria and fungi did not differ significantly among different aggregate sizes either under RS or RD (Table 1). But bacterial diversity was higher under RS compared to RD both within bulk soil and four sizes of aggregates (*p* < 0.05, Table 1, Figure 2A). In contrast, fungi showed opposite patterns except for 2–5 mm fraction (*p* < 0.05, Table 1; Figure 2B).

PCoA based on the Bray–Curtis dissimilarity and PERMANOVA revealed that changes in aggregate size and cropping system significantly influenced the compositions of bacterial and fungal communities (Table 1; Figures 2C,D). In addition, bacterial community differed between RS and RD within bulk soil and four fractions of aggregates (Supplementary Table S1). Nevertheless, only fungal community associated with 0.25-2 mm fraction significantly differed between RS and RD (Supplementary Table S1).

### Bacterial and fungal assemblages

**Figure 3:**
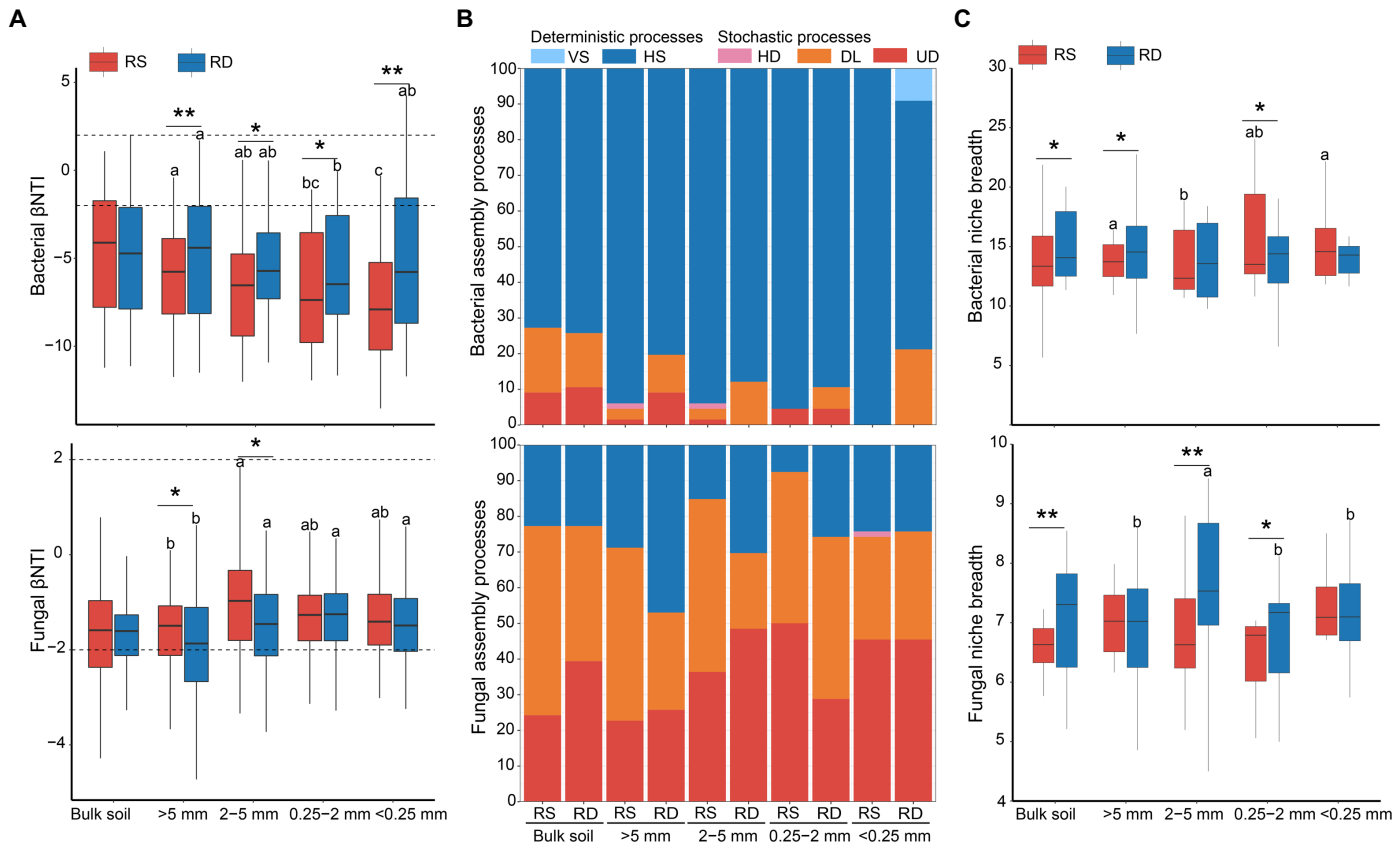
The ecological processes of microbial assemblages in soil aggregates under single-season (RS) and double-season (RD) cropping systems. **(A)** Comparison of the beta-nearest taxon index (βNTI) of bacterial and fungal communities among four aggregate sizes. **(B)** Relative contributions of assembly processes in shaping communities within soil aggregates. *VS*: variable selection; HS: homogeneous selection; HD: homogenizing dispersal; DL: dispersal limitation; UD: undominated processes. **(C)** Niche breadth of bacterial and fungal communities among aggregate sizes under single-season and double-season rice cropping systems. Significant differences at *p* < 0.05 showed only. The asterisks indicate a significant difference between the two cropping systems (*p < 0.05, **p < 0.01). Whereas different letters indicate significant differences among different aggregate sizes.

Assemblage of bacteria was dominated by deterministic processes of homogeneous selection (Figures 3A,B) and was significantly affected by aggregate size (R^2^ = 0.097, *p* < 0.01) and cropping system (*R*^2^ = 0.043, *p* < 0.01, Table 1). The proportion of homogeneous selection increased slightly with the decrease of aggregate sizes under both RS and RD, peaking within <0.25 mm (100%) and 0.25–2 mm (89.39%) fractions, respectively (Figure 3B). Although the ratio of homogeneous selection in bulk soil did not differ between RS and RD, the impact of the two cropping systems on bacterial assemblage was most pronounced within <0.25 mm aggregates. Specifically, the ratio of homogenous selection decreased by 13.64, 6.06, 6.06 and 30.30% within >5 mm, 2–5 mm, 0.25–2 mm and < 0.25 mm aggregates under RD compared to RS, respectively (Figure 3B).

Unlike bacteria, the fungal assemblage was governed by stochastic processes of dispersal limitation and undominated processes (Figures 3A,B) but was not significantly impacted by aggregate size (*R*^2^ = 0.019, *p* > 0.05) and cropping system (*R*^2^ = 0.014, *p* > 0.05, Table 1). The percentage of dispersal limitation was lowest within <0.25 mm fraction (28.79%) under RS, while lowest within 2–5 mm (21.21%) under RD. The percentage of undominated processes was lowest within >5 mm (22.73%) than other aggregates under RS, while lowest within 0.25–2 mm (28.79%) under RD. In addition, the importance of dispersal limitation under RD was reduced by 8.38, 27.55 and 27.27%, respectively, within bulk soil, >5 mm and 2–5 mm aggregates compared to RS. In comparison, the importance of undominated processes increased by 4.33, 20.30 and 12.12%. However, for 0.25–2 mm aggregate, the importance of dispersal limitation was increased by 3.03% under RD compared to RS, and the importance of undominated processes was reduced by 21.21%. In total, the impact of RS and RD on fungal assemblage was mainly reflected in >5 mm, 2–5 mm, and 0.25–2 mm aggregates (Figure 3B).

Niche breadth further identified the contribution of selection and dispersal to microbial communities (Figure 3C). The lowest niche breadth of bacteria was found in 2–5 mm aggregate under RS, while no significant difference was observed among aggregate sizes under RD. In addition, bacteria had a broader niche under RD than under RS within bulk soil and aggregates except for <0.25 mm fraction (Figure 3C), which was consistent with the lower deterministic processes under RD compared to RS. No significant differences were discovered among the four aggregate fractions under RS for fungi. The fungal community within 2–5 mm aggregate had the widest niche under RD. Furthermore, the niche breadth of fungi was higher under RD than RS within bulk soil, 2–5 mm, and 0.25–2 mm aggregates.

### Co-occurrence patterns in the microbial networks

**Figure 4:**
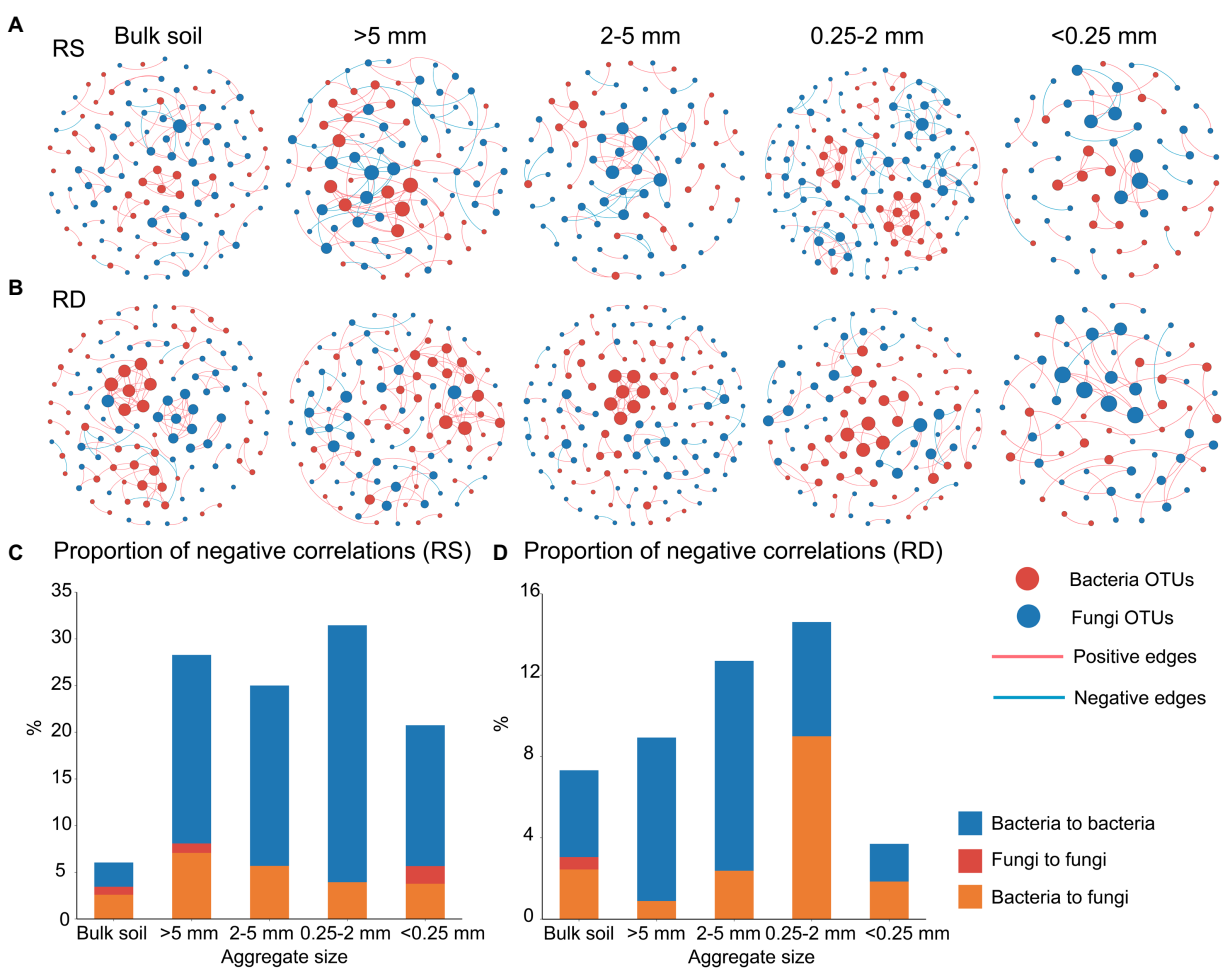
Microbial co-occurrence networks **(A** and **B)**and the proportion of negative correlation (PNC, **C** and **D**) across aggregate sizes under single-season (RS) and double-season (RD) cropping systems.

Co-occurrence network analysis is a powerful way to elucidate the potential roles of microbial interactions in assembly processes. We found that the co-occurrence pattern of microbial communities differed significantly among aggregates (Figure 4). The total number of nodes and edges and average degree were lowest in <0.25 mm aggregate than the other three aggregate networks under both RS and RD (Supplementary Table S2).

As the proportion of negative correlation (PNC) in the microbial networks indicates competition among individuals, it can be inferred that decreased negative microbial relationships may lead to weak selection and low compositional turnover in the microbial communities (Luan et al., 2020). We observed that PNC in overall networks was significantly affected by aggregate size (*R*^2^ = 0.032, *p* < 0.05) and cropping system (*R*^2^ = 0.073, *p* < 0.01, Table 1). Within the bacterial community, a reduced PNC occurred in <0.25 mm fraction compared with the other aggregate fractions under RS or RD (Figures 4C,D). Additionally, the PNC of bacteria within bulk soil was enhanced in RD compared to RS but weakened within the four aggregates (Supplementary Table S2). However, there was no marked variation pattern in PNC within fungal community regardless of aggregate size or cropping system. Notably, PNC of the overall networks was significantly correlated with bacterial assemblage (*R* = 0.050, *p* < 0.01) but not fungi (Table 2).

**Table 2:** Correlation between soil properties, microbial interactions, and bacterial and fungal assemblages (βNTI).

	Bacterial assemblage	Fungal assemblage
***Soil properties***		
TP	0.081^**^	−0.143^**^
SOC	0.032^**^	−0.063^**^
C:N	−0.034^**^	0.042^**^
N:P	−0.061^**^	−0.034^**^
pH	NS	−0.024^*^
TN	NS	NS
*Microbial interactions*		
PNC	0.050**	NS

The asterisks indicate a significant correlation (*p < 0.05, **p < 0.01), NS means no significant difference.

SOC, Soil organic carbon; TN, Total nitrogen; TP, Total phosphorus; C:N, The ratio of soil organic carbon and nitrogen; N:P, The ratio of soil nitrogen and phosphorus; PNC, the proportion of negative correlation.

### Potential importance predictors of bacterial and fungal assemblages and microbial activities

Mantel tests were carried out to reveal the abiotic factors that influence bacterial and fungal assemblages. Soil TP was the most important abiotic factor driving both bacterial (*R* = 0.081, *p* < 0.01) and fungal (*R* = −0.143, *p* < 0.01) assemblages (Table 2). The βNTI for bacteria gradually increased under both RS and RD as TP increased (Supplementary Figure S4A). However, the βNTI for fungi changed from βNTI <2 to βNTI <− 2 as TP concentration increased regardless of cropping system (Supplementary Figure S4B). Bacterial assemblage was also significantly correlated with N:P (*R* = −0.061, *p* < 0.01), SOC (*R* = 0.032, *p* < 0.01) and C:N (*R* = 0.034, p < 0.01). Concerning fungi, we found significant correlations between fungal assemblage and SOC (*R* = −0.063, *p* < 0.01), C:N (R = 0.042, *p* < 0.01), N:P (*R* = −0.034, *p* < 0.01) and pH (*R* = −0.024, *p* < 0.05).

Random forest analysis was performed to separate and assess the important predictors of microbial activities. All measured soil abiotic and biotic variables accounted for 50.12, 49.70 and 35.28% of the variation in C-, N-, and P-cycling, respectively (Supplementary Figure S5). Soil pH and SOC were the most important predictors of soil C-, and N-cycling (Supplementary Figures S5A,B). Furthermore, fungal composition and bacterial assemblage contributed significantly to C-cycling. The compositions and assemblages of bacteria and fungi significantly regulated N-cycling. Additionally, P-cycling was driven by TN, SOC and N:P, and bacterial and fungal compositions and assemblages (Supplementary Figure S5C).

**Figure 5:**
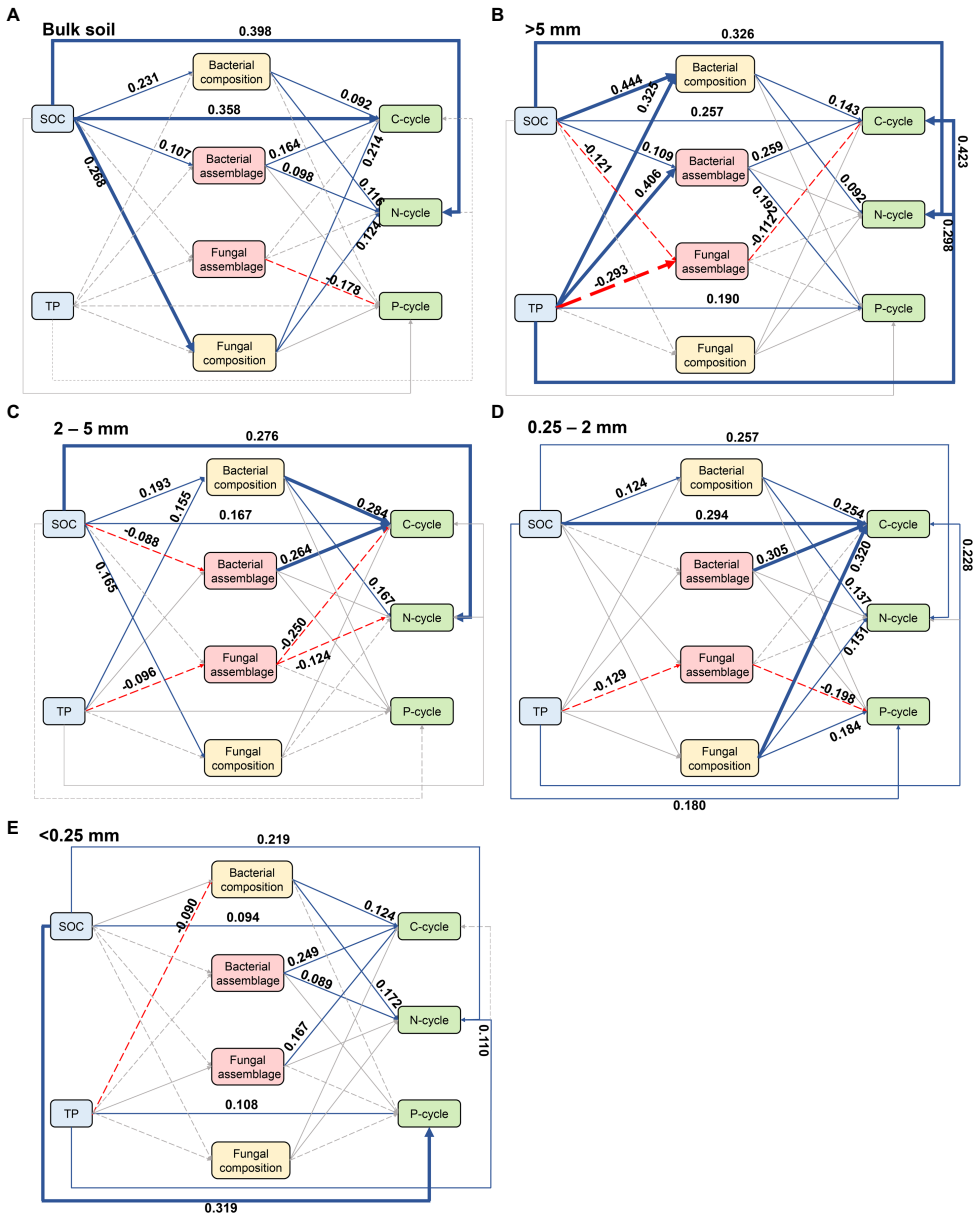
The partial least squares structural equation model (PLS-SEM) showing the effects of soil physicochemical properties and microbial communities on soil nutrient cycling across aggregate sizes. **(A)** Bulk soil, **(B)** >5 mm size, **(C)** 2-5 mm size, **(D)** 0.25-2 mm size, **(E)** <0.25 mm size. Numbers adjacent to the arrows are standardized path coefficients, analogous to relative regression weights and indicative of the effect size of the relationship. Blue solid and red dashed arrows indicate significant positive and negative relationships, respectively. The width of the arrow is proportional to the strength of path coefficients. Paths with non-significant coefficients are presented as gray lines.

PLS-SEM was conducted based on the known effects of soil properties on microbial assemblages and soil nutrient cycling (Figure 5). Both bacterial and fungal assemblages were significantly related to nutrient cycling within bulk soil and all aggregate sizes. While SOC and TP mainly regulated the impact of assembly processes on nutrient cycling in bulk soil, >5 mm, 2-5 mm, and 0.25-2 mm fractions. Specifically, deterministic bacterial assemblage significantly promoted C- and N-cycling within bulk soil (Figure 5A), as well as C-cycling and P-cycling within >5 mm aggregate (Figure 5B) under the influence of SOC. The stochastic fungal assemblage was affected by SOC and significantly suppressed C-cycling within >5 mm aggregate (Figure 5B). TP drove the stochastic processes of fungi, thus inhibiting the C-and N-cycling within 2–5 mm aggregate (Figure 5C) and P-cycling within 0.25–2 mm aggregate (Figure 5D), respectively. However, deterministic processes of bacteria showed positive covariation with C-cycling within 0.25–2 mm aggregate (Figure 5D) and C-cycling as well as N-cycling within <0.25 mm aggregate (Figure 5E), respectively. Fungal assemblage by stochastic processes exhibited a direct relationship with P-cycling in bulk soil and with C-cycling in <0.25 mm aggregate (Figure 5E).

## Discussion

### Deterministic processes of bacteria and fungi were enhanced within <0.25 mm aggregate

The ecological pattern of microbial communities in soil aggregates has received little attention so far (Wilpiszeski et al., 2019). We observed that deterministic processes of homogeneous selection were the main drivers for bacterial community, consistent with the results of most microbial assemblages studies that niche-based processes generally impact bacteria more than neutral processes (Powell et al., 2015; Delgado-Baquerizo et al., 2018; Wang et al., 2020; Xue et al., 2021; Zhang et al., 2022). Contrarily, other works have shown that bacterial assemblage is more stochastic in agricultural soil (Chen et al., 2020; Li et al., 2021). The discrepancy in outcomes is likely related to the spatial scales. As pointed out in these studies, stochastic processes govern bacterial assemblage in local scales with less environmental variation and the species pools are characterized by ecological generalists. In contrast, the importance of determinism to the overall structure of bacteria increased at regional or centimeter scales. Our results provide evidence that bacterial assemblage is predominantly deterministic at aggregate scales, further confirming that distinct assembly processes alter the community fundamentally and depend on the scale at which communities are investigated. As a result, more dimensions should be considered when linking the relative effects of ecological processes and microbial assemblages.

In addition, we found that the relative contribution of deterministic bacterial assemblage increased within microaggregates (<0.25 mm fraction) compared to macroaggregates (>5 mm, 2–5 mm and 0.25-2 mm fractions). Previous studies observed that microaggregates have more recalcitrant organic matter and lower labile carbon than macroaggregates (Trivedi et al., 2017; Wang et al., 2021). Thus, limited substrates within microaggregates enhanced deterministic processes by exerting selective stress on microbial survival and growth. Consistent with this idea, we found an enrichment of *Alphaproteobacteria* within microaggregates, which are reported to be tolerant to oligotrophic conditions (Zheng et al., 2021).

Unlike the situation for bacteria, stochastic processes, especially dispersal limitation and undominated processes, were the primary contributors to fungal turnover within soil aggregates. Recent studies on other scales also found a lack of significant selection effects on fungal community (Powell et al., 2015; Gao et al., 2020; Huang et al., 2022). One possible explanation might be that the fungal hyphal network facilitates water and nutrient uptake, which confers excellent resistance (Yuste et al., 2011; Barnard et al., 2013). Moreover, we identified a weakened stochastic fungal assemblage in microaggregates relative to that in macroaggregates, which likely supports the idea that microaggregates might be less prone to stochastic processes caused by either random dispersal or ecological disturbance (Dong et al., 2021). In short, our results support the contribution of deterministic processes to microbial assemblages within microaggregates being greater than macroaggregates.

We also confirmed that different cropping systems significantly impact bacterial assemblage. Compared with the single-season (RS), the contribution of deterministic processes to bacterial assemblage was reduced by double-season cropping system (RD) within both bulk soil and across aggregate sizes. Dramatic increases in SOC and TN under RD were observed, indicating that increases in soil nutrients may attenuate determinism by alleviating selective pressure for bacterial survival and fitness in nutrient-rich soil (Zhang et al., 2022). Regarding biotic factors, we found lower PNC within bacteria in RD, suggesting weaker selection due to reduced competition between microbes. Unlike bacteria, the stochastic processes were less dominant for fungal assemblage under RD than RS. This was supported by a previous report that stochasticity was less critical to microbial assemblages as resource availability increased (Chase, 2010). Collectively, these findings indicate that different cropping systems may impact the balance of deterministic and stochastic processes and contribute to the establishment of a functional equilibrium within microbial assemblages.

### Changes in total phosphorus content and microbial interactions drive microbial assemblages

Knowledge of the underlying factors that affect microbial assemblages is essential for a comprehensive understanding of microbial ecology (Zhao et al., 2022). Several studies have shown that soil abiotic factors such as temperature (He et al., 2021) and pH (Tripathi et al., 2018), as well as organic matter (Dini-Andreote et al., 2015) are essential to microbial assemblages. And, our study reveals that total soil phosphorus (TP) has a very significant association with bacterial and fungal assemblages, indicating that TP is a primary factor mediating the balance of deterministic and stochastic processes for microbial communities in agricultural soil at aggregate scales. However, divergences in TP resulted in a change in bacterial community from homogeneous selection to stochasticity. Fungal assemblage gradually shifted from stochasticity to homogeneous selection. Similarly, the same soil properties were found to cause distinct responses in the assembly mechanisms of abundant and rare species in forest and agricultural fields (Jiao and Lu, 2020; Peng et al., 2021). These results suggest that different microbial taxa have assembly strategies that are distinct from changes in the same soil variables, and that studies exploring the response of microbial assemblages to soil abiotic factors, e.g., ORP, are needed in the future.

Microbial interaction networks offer critical insight into factors that mediate the assembly of microbial communities (He et al., 2021). Our study showed a direct and strong positive correlation between PNC and bacterial βNTI. Previous findings confirmed that competitive interaction might be essential in driving deterministic microbial assemblages (Meuser et al., 2013; Goberna et al., 2014). This might likely be linked to the opinion that competitive interaction caused by limited nutrient sources and antagonistic effects among species would constrain the coexistence of species (Becker et al., 2012; Maynard et al., 2017; Li et al., 2020), which led to substantial selection and high compositional turnover in the microbial communities (Bahram et al., 2018; Luan et al., 2020).

### Inconsistent effects of deterministic bacterial assemblage and stochastic fungal assemblage on nutrient cycling in soil aggregates

This study also illustrates that bacterial and fungal assemblies are essential predictors of microbial activities and significantly correlate with C-, N-, and P-cycling in the agroecosystem. Furthermore, the impacts of microbial assemblages on soil nutrient cycling are aggregate-specific, supporting a relationship between effect of microbial assemblages on nutrient cycling and aggregate size.

The assembly processes impose constraints on community membership and subsequently determine microbial activities (Stegen et al., 2016). Under deterministic selection, the success or failure of colonization in each ecosystem is based on how well the functional traits align with environmental conditions (Bao et al., 2020). We found that deterministic bacterial assemblage was significantly and positively correlated with nutrient cycling within all aggregate sizes, suggesting that microbial metabolic capability may be facilitated by the deterministic assembly of bacteria taxa well-adapted to aggregates (Graham and Stegen, 2017). In contrast to determinism, stochasticity can result in more diversified community structures but at the cost of suppressing functional traits of microorganisms (Knelman and Nemergut, 2014). Consistent with this influence of stochasticity, we observed that the increase in soil nutrients was associated with a reduced contribution of stochastic processes to microbes, which was supported by the notion that stochasticity-based microbial assemblages decrease biogeochemical functions (Graham and Stegen, 2017; Luan et al., 2020).

Additionally, we found that the impacts of microbial assemblages on C-, N-, and P-cycling were more susceptible to physicochemical properties of agricultural fields (e.g., SOC and TP) in macroaggregates. The fresh organic matter from farmland crops first enters the macroaggregates and disturbs the microorganisms associated with large fractions (Six et al., 1999). Such disturbance might impact the assembly processes of original microbial communities, thereby influencing microbial diversity and composition, with downstream impacts on the function of ecosystem. In contrast, residual organic substances circulate into microaggregates, which form recalcitrant components that are not conducive to microbial utilization through mineral binding (e.g., adsorption) (Trivedi et al., 2017). In addition, microaggregates have been demonstrated to provide stronger physical protection to microorganisms than macroaggregates (Tian et al., 2021) and might avoid the influence of environmental disturbance. Overall, our findings emphasize the critical role of maintaining aggregate stability in facilitating ecosystem services by coupling microbial assemblages with soil nutrient cycling.

## Conclusion

**Figure 6:**
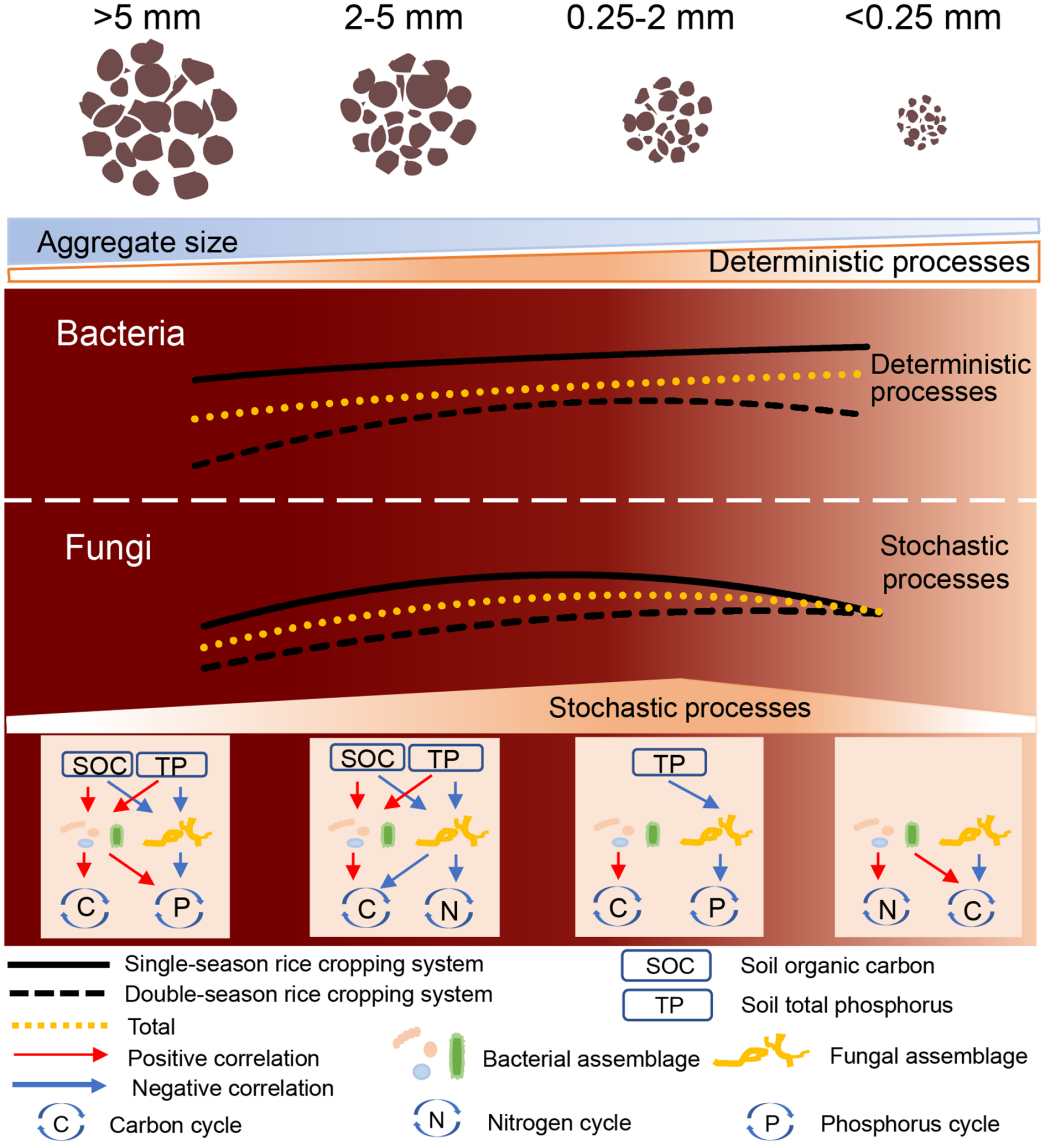
A conceptual paradigm elucidating the bacterial and fungal assemblages and their associations with soil carbon, nitrogen, as well as phosphorus cycling within different aggregate sizes fractions in agricultural soil.

In light of the information obtained from this study, we propose the model depicted in Figure 6. In this model, the proportion of stochastic and deterministic processes that underpin assemblages of bacterial and fungal communities are directly related to soil function across the four aggregate sizes in a typical agroecosystem. Selection for homogenous bacterial assemblage increases with decreasing aggregate size. In contrast, fungal assemblage is dominated by dispersal limitation and undominated processes, and the importance of stochastic is lowest within microaggregates (<0.25 mm). Soil TP and microbial interactions jointly influence microbial assemblages, particularly in bacterial community. Furthermore, microbial assemblages are strongly associated with soil functions within all sizes of aggregate, and aggregate properties mainly regulate effects of assembly mechanism on nutrient cycling within macroaggregates (>0.25 mm). These results offer new insights into understanding the response of microbial assemblages to perturbations and environmental changes in agricultural soils at the aggregate scales, and further validate the linkages between microbial assemblages and ecosystem functions.

## Data availability statement

The datasets presented in this study can be found in online repositories. The names of the repository/repositories and accession number(s) can be found in the article/Supplementary material.

## Author contributions

SZ: conceptualization, methodology, data curation, formal analysis, visualization, and writing – original draft. WH: investigation and formal analysis. YX: investigation and revision. HZ: data curation and visualization. ZK: revision and supervision. LW: writing – review, editing, funding acquisition, and supervision. All authors contributed to the article and approved the submitted version.

## Funding

This research was funded by the National Natural Science Foundation of China (Grant Nos. 31971470, 31660149, and 31560143), the National Key R & D Program of China (Grant No. 2019YFC0605005), the Key Natural Science Foundation of Jiangxi, China (Grant No. 20212ACB205009), the Natural Science Foundation of Jiangxi Province, China (Grant No. 20202BAB203025) and Youth Science Fund Project in Jiangxi Province (Grant No. 2018BA214004).

## Conflict of interest

The authors declare that the research was conducted in the absence of any commercial or financial relationships that could be construed as a potential conflict of interest.

## Publisher’s note

All claims expressed in this article are solely those of the authors and do not necessarily represent those of their affiliated organizations, or those of the publisher, the editors and the reviewers. Any product that may be evaluated in this article, or claim that may be made by its manufacturer, is not guaranteed or endorsed by the publisher.

## Supplementary material

The Supplementary material for this article can be found online at: https://www.frontiersin.org/articles/10.3389/fmicb.2022.1038536/full#supplementary-material
