# Supplementary material for: Linking bacterial and fungal assemblages to soil nutrient cycling within different aggregate sizes in agroecosystem
Source: Front Microbiol. 2022 Nov 14;13:1038536. doi: 10.3389/fmicb.2022.1038536 (PMC9701741; doi:10.3389/fmicb.2022.1038536)
Supplement: Supplementary file 1 [file Table_1.DOCX]

**Supplementary Table S1.** Permutational multivariate ANOVA showing differences of bacterial and fungal communities between single-season (RS) and double-season (RD) cropping systems across bulk soil and four aggregate sizes.

|  |  | | Bacteria | | | Fungi | |
| --- | --- | --- | --- | --- | --- | --- | --- |
|  | |  | | F | R^2^ | F | R^2^ |
| Bulk soil | | RS-RD | | 1.782 | 0.075* | 1.212 | 0.052 |
| >5 mm | | RS-RD | | 2.134 | 0.088** | 1.030 | 0.045 |
| 2-5 mm | | RS-RD | | 2.356 | 0.097** | 1.026 | 0.045 |
| 0.25-2 mm | | RS-RD | | 2.198 | 0.091** | 1.512 | 0.064* |
| <0.25 mm | | RS-RD | | 2.021 | 0.084** | 1.187 | 0.052 |

The asterisks indicate a significant influence (* p < 0.05, ** p < 0.01).
