# Supplementary material for: Linking bacterial and fungal assemblages to soil nutrient cycling within different aggregate sizes in agroecosystem
Source: Front Microbiol. 2022 Nov 14;13:1038536. doi: 10.3389/fmicb.2022.1038536 (PMC9701741; doi:10.3389/fmicb.2022.1038536)
Supplement: Supplementary file 2 [file Table_2.DOCX]

**Supplementary Table S2**. Topological parameters of co-occurrence networks in different aggregate sizes under single-season and double-season rice cropping systems.

|  | RS | | | | | RD | | | | |
| --- | --- | --- | --- | --- | --- | --- | --- | --- | --- | --- |
|  | Bulk soil | >5 mm | 2-5 mm | 0.25-2 mm | <0.25 mm | Bulk soil | >5 mm | 2-5 mm | 0.25-2 mm | <0.25 mm |
| Number of nodes | 126 | 91 | 90 | 132 | 65 | 125 | 112 | 137 | 108 | 67 |
| Bacterial nodes (%) | 65.87% | 65.93% | 70.00% | 68.18% | 61.54% | 46.40% | 51.79% | 57.66% | 50.00% | 68.66% |
| Fungal nodes (%) | 34.13% | 34.07% | 30.00% | 31.82% | 38.46% | 53.60% | 48.21% | 42.34% | 50.00% | 29.85% |
| Number of edges | 116 | 99 | 88 | 178 | 53 | 164 | 112 | 126 | 89 | 54 |
| Positive edges (%) | 93.97% | 71.72% | 75.00% | 68.54% | 79.25% | 92.68% | 91.07% | 87.30% | 85.39% | 96.30% |
| Negative edges (%) | 6.03% | 28.28% | 25.00% | 31.46% | 20.75% | 7.32% | 8.93% | 12.70% | 14.61% | 3.70% |
| Average degree | 1.84 | 2.18 | 1.96 | 2.70 | 1.63 | 2.62 | 2.00 | 1.84 | 1.65 | 1.61 |
| Network diameter | 10 | 7 | 11 | 10 | 6 | 10 | 6 | 10 | 9 | 5 |
| Modularity | 0.909 | 0.842 | 0.816 | 0.871 | 0.895 | 0.855 | 0.919 | 0.935 | 0.936 | 0.911 |
| Average path coefficient | 0.581 | 0.526 | 0.444 | 0.526 | 0.645 | 0.672 | 0.652 | 0.669 | 0.607 | 0.494 |
| Average path length | 3.395 | 3.139 | 4.082 | 3.936 | 1.724 | 3.668 | 1.905 | 2.589 | 2.513 | 1.674 |

RS: Single-season cropping systems; RD: double-season cropping systems.
