# Supplementary figures and images for: Linking bacterial and fungal assemblages to soil nutrient cycling within different aggregate sizes in agroecosystem
Source: Front Microbiol. 2022 Nov 14;13:1038536. doi: 10.3389/fmicb.2022.1038536 (PMC9701741; doi:10.3389/fmicb.2022.1038536)

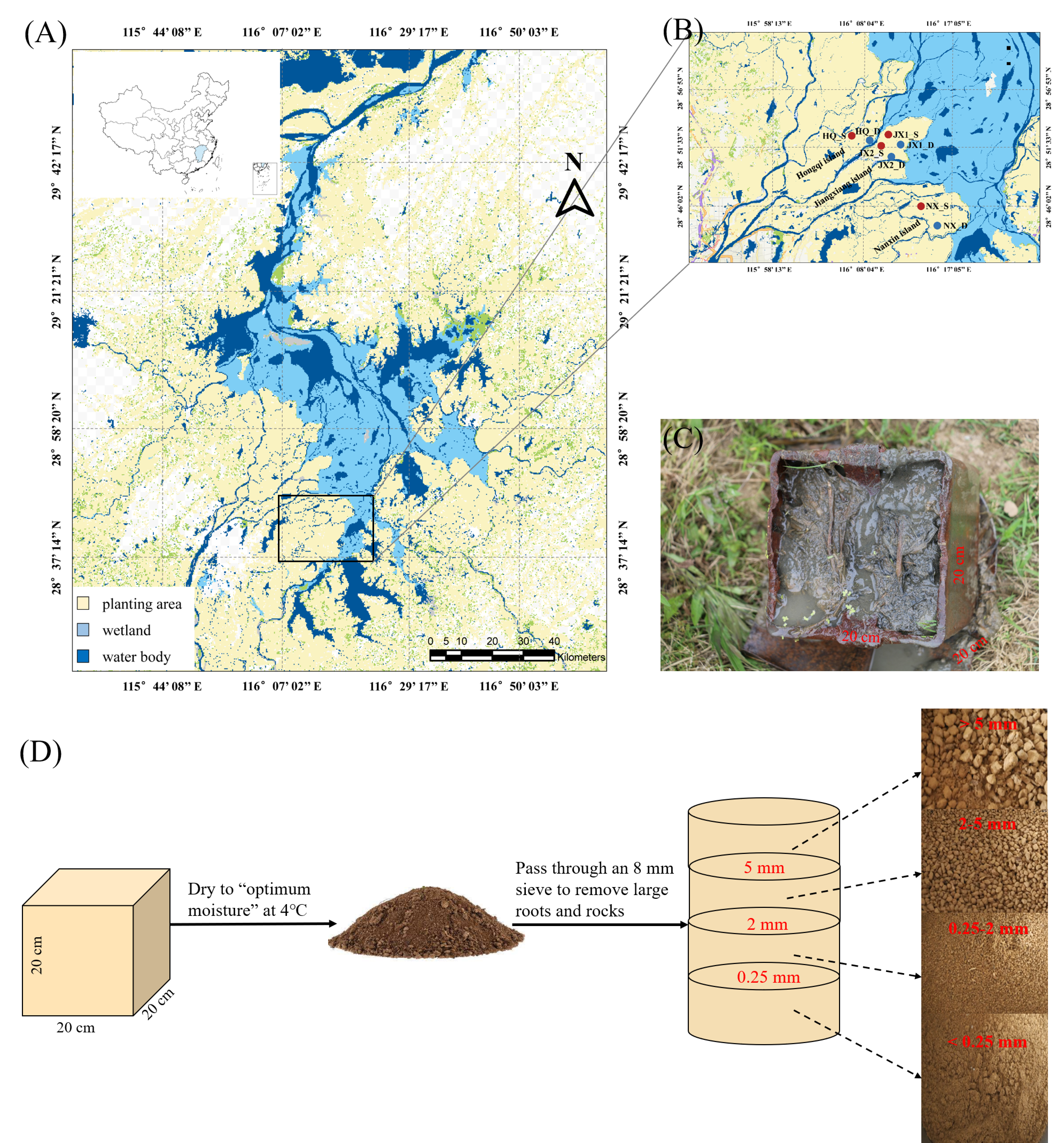

Supplement: Supplementary file 3 [file Image_1.TIF]

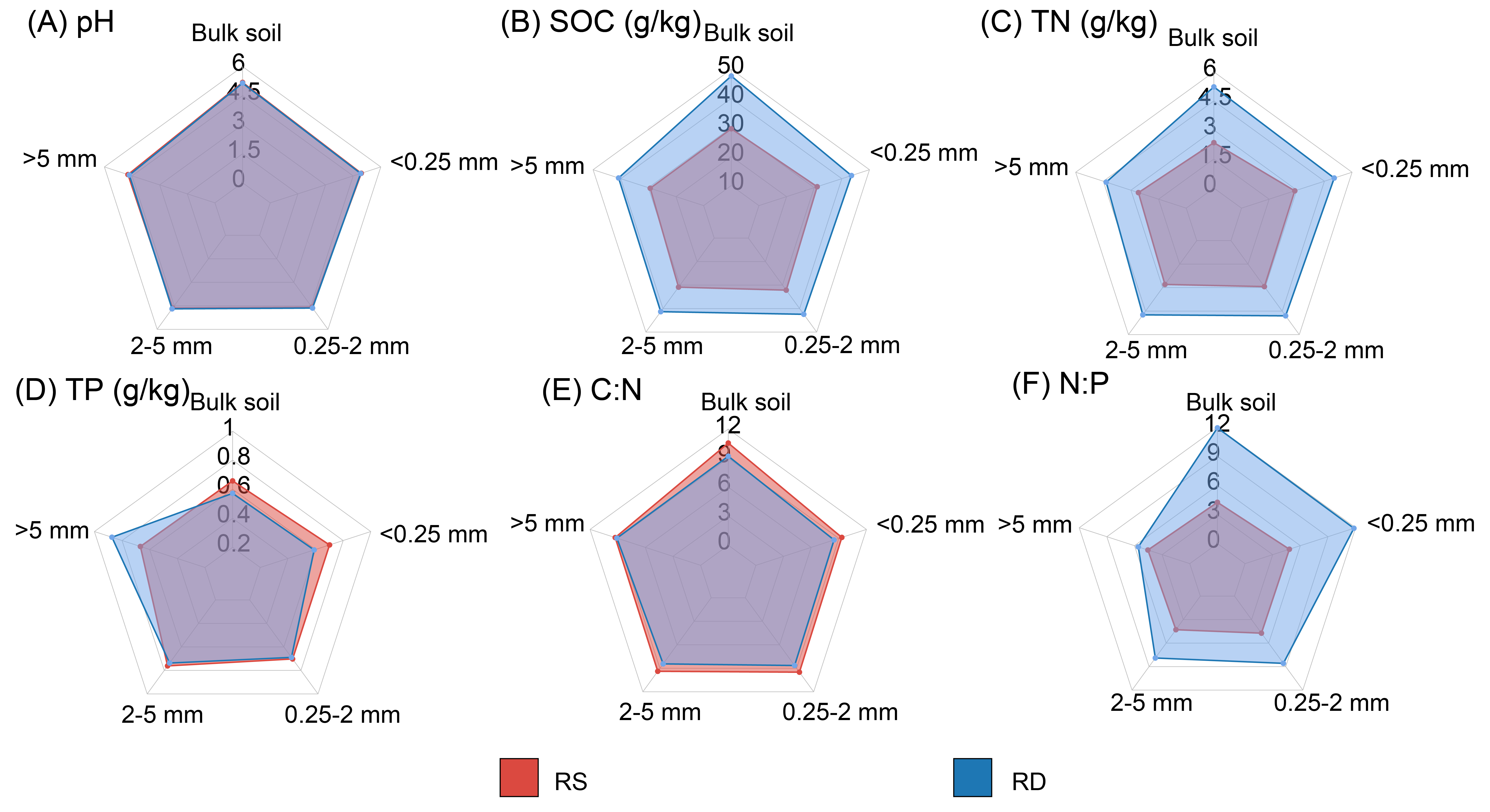

Supplement: Supplementary file 4 [file Image_2.TIF]

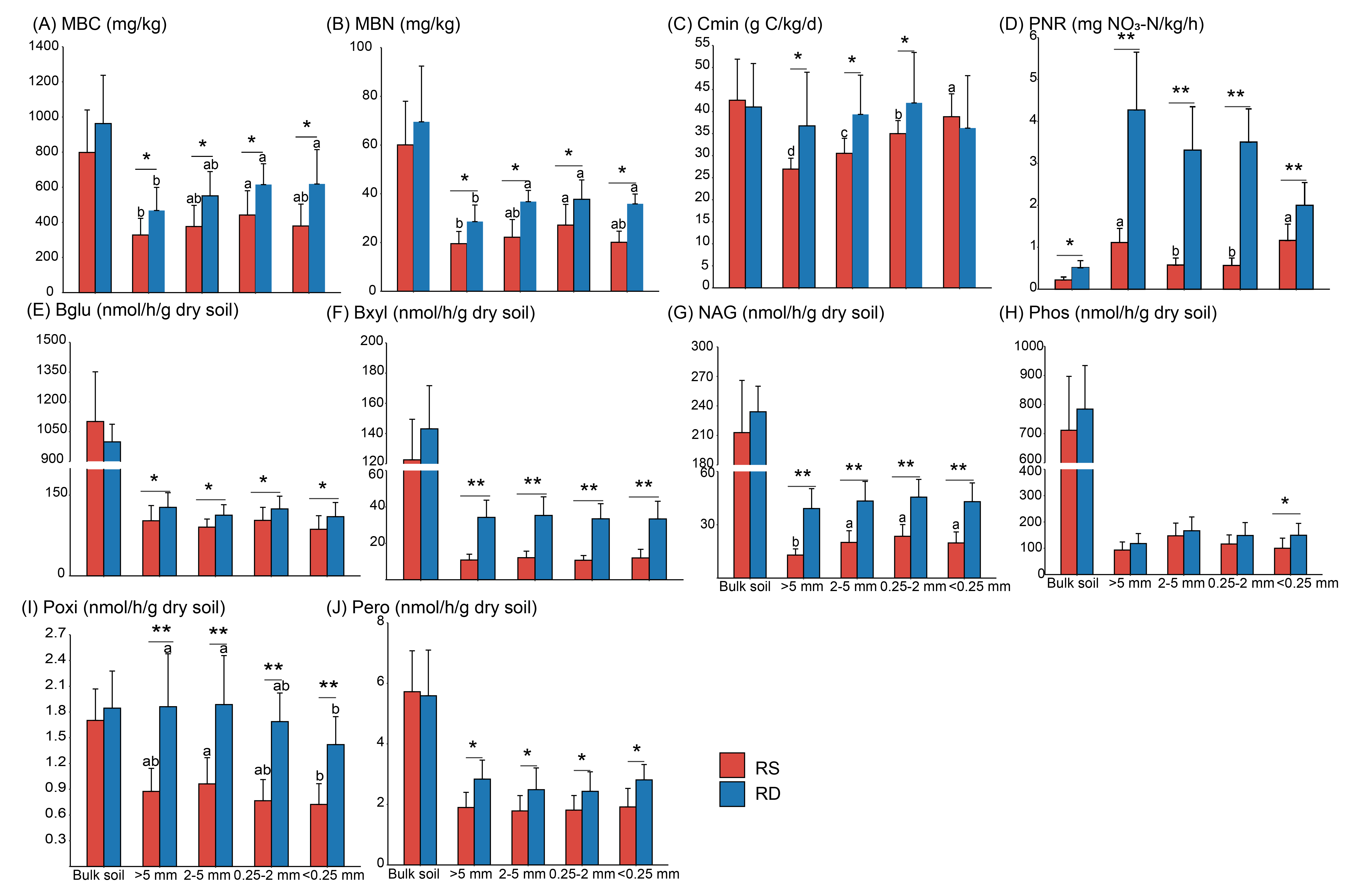

Supplement: Supplementary file 5 [file Image_3.TIF]

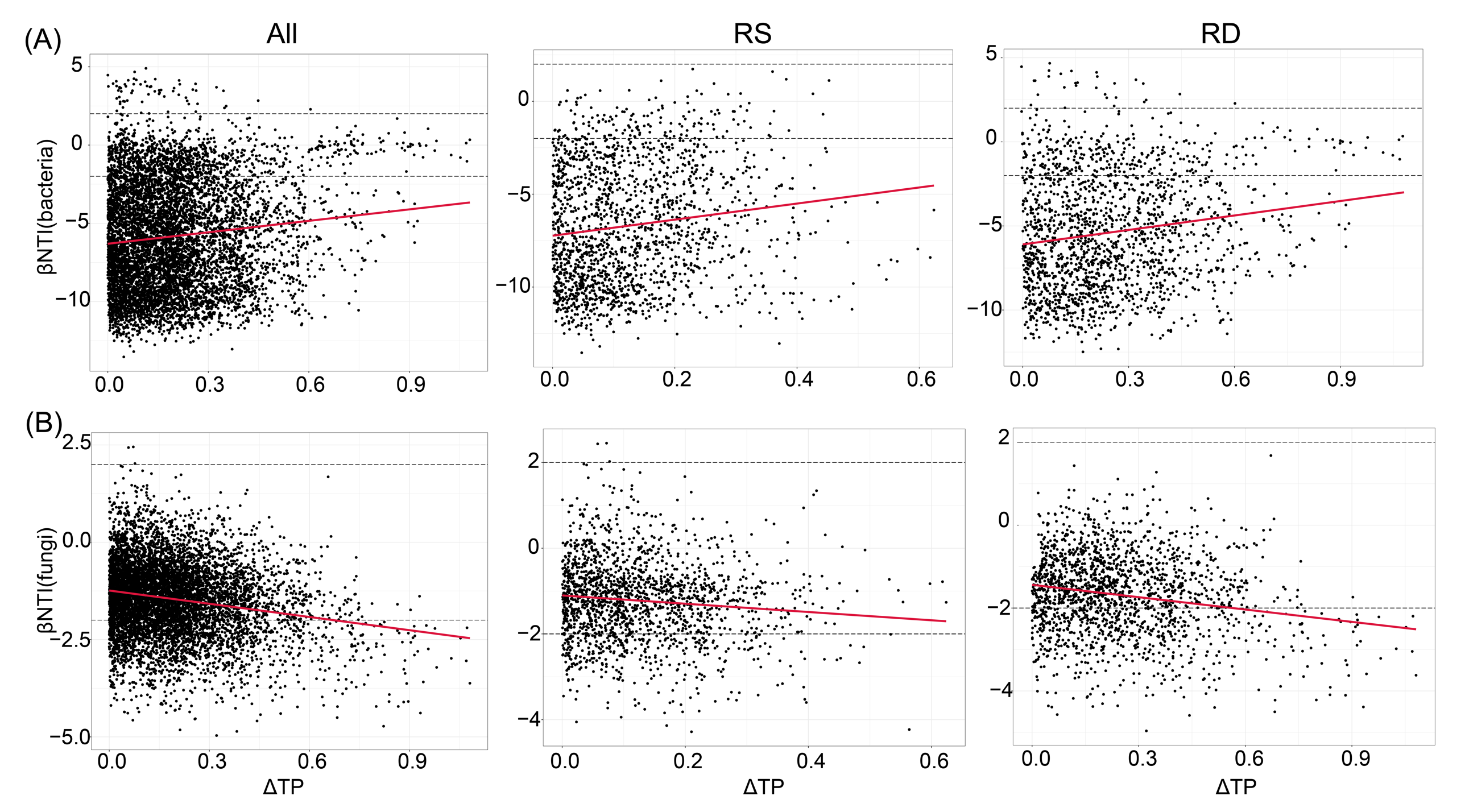

Supplement: Supplementary file 6 [file Image_4.TIF]

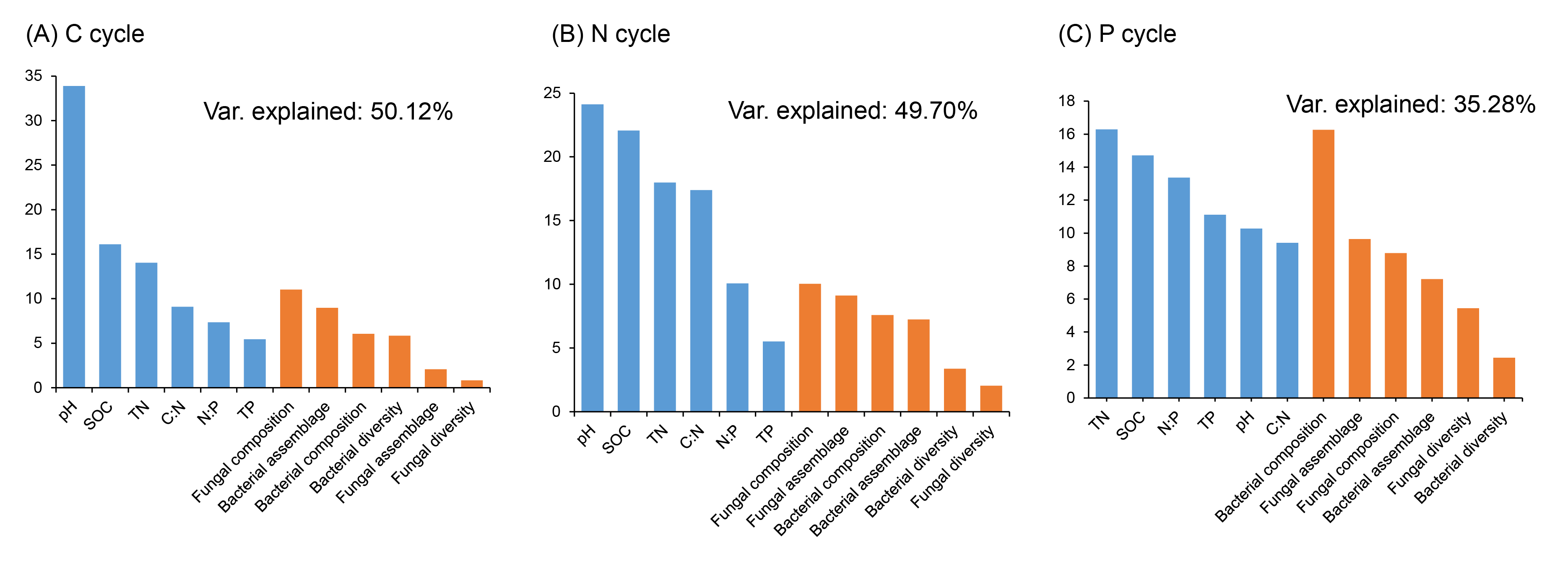

Supplement: Supplementary file 7 [file Image_5.TIF]
